# U1 snRNP-Dependent Suppression of Polyadenylation: Physiological Role and Therapeutic Opportunities in Cancer

**DOI:** 10.1155/2013/846510

**Published:** 2013-10-27

**Authors:** Lee Spraggon, Luca Cartegni

**Affiliations:** ^1^Molecular Pharmacology and Chemistry Program, Memorial Sloan-Kettering Cancer Center, New York, NY 10021, USA; ^2^Department of Chemical Biology, Ernest Mario School of Pharmacy, Rutgers, The State University of New Jersey, Piscataway, NJ 08854, USA

## Abstract

Pre-mRNA splicing and polyadenylation are critical steps in the maturation of eukaryotic mRNA. U1 snRNP is an essential component of the splicing machinery and participates in splice-site selection and spliceosome assembly by base-pairing to the 5′ splice site. U1 snRNP also plays an additional, nonsplicing global function in 3′ end mRNA processing; it actively suppresses the polyadenylation machinery from using early, mostly intronic polyadenylation signals which would lead to aberrant, truncated mRNAs. Thus, U1 snRNP safeguards pre-mRNA transcripts against premature polyadenylation and contributes to the regulation of alternative polyadenylation. Here, we review the role of U1 snRNP in 3′ end mRNA processing, outline the evidence that led to the recognition of its physiological, general role in inhibiting polyadenylation, and finally highlight the possibility of manipulating this U1 snRNP function for therapeutic purposes in cancer.

## 1. Introduction

The generation of translationally competent messenger RNAs (mRNAs) is a complex molecular process that involves distinctive enzymatic reactions and dedicated cellular machineries that result in the splicing, capping, editing, and polyadenylation of a pre-mRNA transcript. During this process, the choice and usage of splice sites (alternative splicing, AS) and of polyadenylation signals (alternative polyadenylation, APA) within a common pre-mRNA can be differentially regulated depending on the developmental state, tissue, and cell type or in response to a variety of physiological stimuli or pathological conditions [1, 2]. Collectively, alternative splicing and polyadenylation are key molecular mechanisms for increasing the functional diversity of the human proteome, allowing the relatively small human genome (<25,000 genes) to generate an excess of 100,000 different protein isoforms [3]. However, because of the pervasiveness and essential role of AS in all physiological processes, aberrant RNA processing is also frequently associated with many diseases [4], and both AS and APA are deregulated and exploited by cancer cells to promote their growth and survival [5–7].

This review will focus on the recently described splicing-independent functions of U1 small ribonucleoprotein particle (snRNP) in pre-mRNA processing, with emphasis on its role in the regulation of APA site selection and in the suppression of intronic polyadenylation (IPA). Furthermore, we will address innovative approaches to leverage U1 snRNP functions as therapeutic avenues in cancer treatment.

## 2. U1 snRNP Canonical Role in Splicing: A Harbinger of Spliceosome Assembly

The established function of U1 snRNP, which includes the 164 nt U1 snRNA, seven Sm proteins, and three U1-specific proteins (U1-70K, U1-A, and U1-C), is its role in the early steps of pre-mRNA splicing as a key component of the spliceosome, the large ribonucleoprotein complex responsible for the removal of intronic sequences and subsequent rejoining of exons, to form a mature mRNA [8].

**Figure 1:**
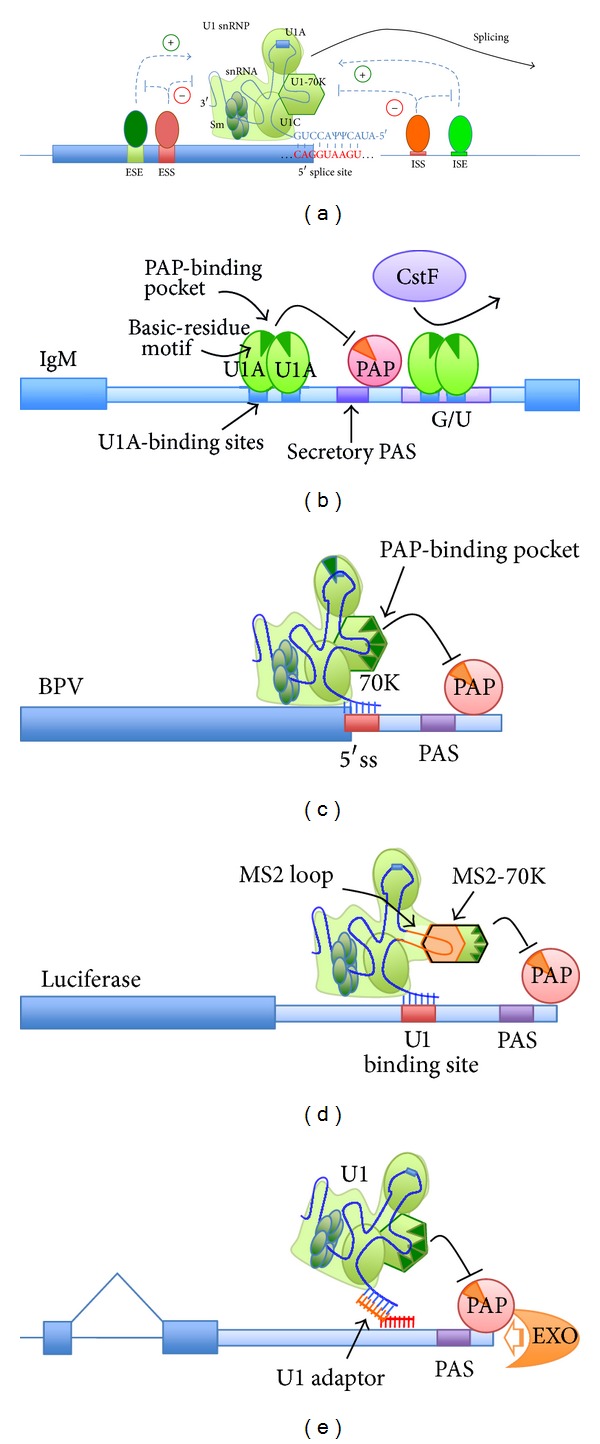
Modes of U1 snRNP activity in splicing and suppression of 3′ end processing. (a) Role of U1 snRNP in splicing. The 5′ end of U1 snRNA base-pairs to the 5′ splice site cotranscriptionally, to define the functional splice donor site. The process is positively and negatively modulated by splicing factors binding to exonic and intronic splicing enhancer and silencers (ESE, ISE, ESS, and ISS, resp.). (b) Role of U1A protein in suppression of IgM cleavage and polyadenylation. U1 snRNP component U1A binds to multiple motifs (blue boxes) upstream or downstream of the intronic PAS (purple box) in IgM C*μ*4 = M1 intron. These sites contain the A(U/G)GCN_1−3_C consensus motif and are similar to U1A-binding site on U1 snRNA. From the upstream sites, U1A multimers interact directly with the C-terminal domain of PAP through a binding pocket formed by the basic-residue motifs (green triangles), blocking polyadenylation. When U1A binds downstream, it prevents CstF binding to the G/U element (light purple box) and thus interferes with cleavage. (c) Role of U1 snRNP in suppression of BPV polyadenylation. U1 binds to a 5′  ss a few nucleotides upstream of the alternative PAS. Like U1A multimers, U1-70K interacts directly with the C-terminal domain of PAP through a binding pocket formed by the basic-residue motifs, blocking polyadenylation. (d) Tethered U1-70 K suppresses polyadenylation. A modified U1-MS2 snRNA is engineered to contain an MS2-binding loop in place of the U1-70 K binding loop. A U1-binding site represses polyadenylation and expression from a luciferase reporter vector when the wt U1 snRNA is expressed. The mutant U1-MS2 snRNA recapitulates PAP inhibition only when a fusion MS2-70 K protein is also coexpressed. (e) U1 adaptors recruit U1 snRNP to suppress polyadenylation. Single-stranded, bifunctional modified ASOs are used to recruit endogenous U1 to target sites within ~1 Kb region upstream of a PAS. The targeting moiety (red) base-pairs to a unique, transcript-specific sequence, while the recruiting moiety contains a consensus 5′  ss sequence to bind U1 snRNP with high affinity. The tethered U1 snRNP suppresses polyadenylation and the mRNA is then degraded by the 3′-5′ exosome. Large blue boxes represent exons/coding regions.

The spliceosome assembles through the sequential binding of the five snRNPs (U1, U2, U4, U5, and U6) and multiple auxiliary RNA-binding proteins to form the large, active spliceosome [8]. U1 snRNP plays an essential role in this process by driving the initial steps of spliceosome assembly onto the pre-mRNA at the exon-intron boundary, through definition of the 5′ splice site (ss) by RNA-RNA base-pairing with the 5′ end of U1 snRNA (Figure 1(a)), which can occur in multiple registers [9]. The 3′ ss and the branch point (BP) are recognized by the U2 complex to form the prespliceosomal complex, and the tri-snRNP complex—containing U4, U5, and U6—is then recruited, with U6 replacing U1 at the 5′ ss. Following the release of U4 and further remodeling in the activated spliceosome, two subsequent transesterification reactions occur, to produce the final spliced exons and the released lariat [8]. Most splicing events, as well as polyadenylation, occur cotranscriptionally, and the integration of the three processes, which mutually affect each other, is in large part mediated by the C-terminal domain (CTD) of the elongating RNA Polymerase II (RNAPII) [2]. Multiple splicing and cleavage/polyadenylation factors, including U1 snRNP, are directly associated with the RNAPII CTD from the onset of transcription and are then deposited on their cognate-binding sites along the pre-mRNA, determining splice-site and poly(A) signal (PAS) selection.

Variants to this main pathway exist and in some cases U1 snRNP might not be strictly required [8, 9], whereas in other situations multiple U1 snRNPs can bind to competing alternative 5′ splice sites [9]. However, the mechanics of the canonical splicing reaction mandate that all snRNPs participate in 1 : 1 stoichiometric ratios in the actual removal of each intron. Hence, it has been a long-standing puzzling observation that the cellular levels of U1 snRNP exceed those of other snRNPs by 2-3-fold [10, 11], suggesting that U1 may carry out additional roles besides its canonical one in splicing. Indeed, its involvement in different aspects of 3′ pre-mRNA end formation has been long proposed [12, 13].

## 3. Alternative 3′ End Processing: Physiological Regulation and Deregulation in Cancer

RNAPII mRNAs in eukaryotic cells undergo 3′ end processing, which typically involves the cotranscriptional endonucleolytic cleavage of the pre-mRNA, followed by the addition of a polyadenylate (poly(A)) tail (reviewed in [7, 14]) by a multisubunit complex that includes CPSF (cleavage and polyadenylation specificity factor), CstF (cleavage stimulation factor), CFI and CFII (cleavage factors I and II), and poly(A) polymerase (PAP). CPSF recognizes the canonical hexanucleotide PAS located upstream of the cleavage site, whilst CstF binds to a less well-defined downstream U/GU-rich region. Following cleavage, PAP promotes polyadenylation of the mRNA, adding the ~250 nt poly(A) tail [14].

Global analysis indicates that approximately 50% of human genes use different PAS, a process known as alternative polyadenylation (APA) [15, 16]. The study of APA has been typically focused towards the multiple PAS located within the same 3′ UTRs (tandem PAS). The choice between tandem PAS can directly affect the levels of gene expression. For example, it can lead to the exclusion in the shorter UTRs of miRNA-binding sites that affect translation and/or of other posttranscriptional regulatory sequences embedded in the 3′ UTR that might influence stability or localization.

Recent transcriptome-wide studies of APA have also highlighted the common occurrence of IPA, with active intronic PAS that are used in up to 20% of human genes, either by read-through into the intron or by usage of an alternative terminal exon within introns [17, 18]. Whereas tandem APA only affects expression levels, usage of these IPA sites leads to shortened mRNAs containing truncated open reading frames (ORFs) and hence expressing qualitatively different protein isoforms, with unique C-terminal domains. The function of these isoforms can be vastly different to their full-length counterparts and often encode for variants with dominant-negative properties [7, 19].

Both APA phenomena—IPA and 3′ UTR variability—add an extra layer of complexity to the genome, by increasing the potential number of transcripts encoded by a single gene and controlling their expression. The mechanisms governing APA have only recently begun to be understood. Variations in the levels of the core polyadenylation factors or abundance of a broad range of transacting RNA-binding proteins have been shown to modulate PAS selection (reviewed in [7]). In addition, emerging evidence suggests that APA is also influenced by chromatin organization and epigenetic modifications at the DNA level [7, 20].

Specific and global modulation of APA are important aspects of many physiological processes, and their deregulation can contribute to the etiology of numerous diseases, including cancer. APA is associated with increased cellular proliferation and potentially with oncogenic transformation, with the switch toward isoforms with a shorter 3′ UTR in proliferating/cancer cells [21–24]. This shift typically removes negative posttranscriptional regulatory motifs (such as miRNA-binding sites), resulting in higher expression levels, for example, of oncogenes.

In addition to global 3′ UTR shortening, activation of specific IPA sites can also be modulated in cancer cells, with the generation of truncated variants possessing oncogenic properties or the suppression in tumors of antitumorigenic variants. For example, the usage of an IPA site in cyclin D1, which normally controls progression through the cell cycle, results in the transforming, constitutively active truncated cyclin D1b isoform [25]. Conversely, a soluble extracellular variant of VEGFR2, which acts as a natural inhibitor of VEGF signaling in lymphangiogenesis [26], is significantly downregulated in neuroblastoma [27].

Yet, despite a greater appreciation of APA-mediated events in cancer, the underlying mechanisms regulating this process remain relatively obscure. One simple possibility is that the increased proliferative rate and transcriptional status of cancer cells might be associated with the general upregulation of the cleavage and polyadenylation machinery. This would lead to a more effective recognition of all PAS, which would then by default result in usage of the more proximal sites because of the directionality of transcription. However, as mentioned, multiple transacting RNA-binding proteins such as CPEB1 [28], hnRNP H [29], and PTB [30, 31] can also affect APA, and their activity might be differentially affected in cancer cells (reviewed in [7, 18]). While future work will be required to delineate the contribution of each of these processes to APA regulation, an intriguing possibility is that the modulation of the levels of U1 snRNP would differentially impact APA in cancer cells and thus contribute to UTR shortening (see below).

## 4. U1 snRNP and Its Role in the Suppression of 3′ End Processing

A putative role for U1 snRNP in mRNA 3′ end processing had been first recognized nearly 30 years ago when it was observed that antibodies directed against U1 snRNP were capable of interfering with *in vitro* polyadenylation reactions [32–34].

These initial observations were followed by reports that the human U1 snRNP-associated protein U1A was capable of inhibiting polyadenylation of its own pre-mRNA by binding two highly conserved U1-snRNA-like motifs in close proximity to the U1A mRNA PAS, *via* direct inhibition of poly(A) polymerase activity (PAP) [13, 35, 36]. U1A has also been implicated in the regulation of an APA switch from membrane-bound to secretory forms of immunoglobulin M (IgM) heavy chain mRNAs [37]. The transition to secretory B cells is associated with the usage of an upstream IPA site, resulting in the truncation of the IgM pre-mRNA, with loss of the membrane-anchor encoding terminal exons. Two separate mechanisms appear to be involved in the regulation of the secretory IPA site (Figure 1(b)). On one hand, U1A binds to multiple U1-snRNA-like A(U/G)GCN_1−3_C-binding sites *upstream* of the PAS and suppresses polyadenylation initiation, by direct inhibitory interactions with PAP, much like the self-regulation of U1A own pre-mRNA [37]. However, U1A also binds to similar tandem U1-snRNA-like motifs *downstream* of the secretory IPA site, positioned between two GU-rich domains normally utilized by the cleavage component CstF64. This impedes CstF64 binding and thus prevents cleavage of the pre-mRNA at the secretory IPA site [38].

In both cases, it is the free, non-snRNP-bound U1A that suppresses PAS usage by either inhibiting formation of the cleavage complex from a downstream position, or by directly repressing PAP activity from the upstream sites. As such, the physiologically decreasing levels of free U1A during B-cell differentiation release the suppression of the IPA site, allowing expression of the secreted IgM ([39]). Importantly, free U1A needs to be present at least in dimer form in order for its single basic-residue motif to form a binding pocket that can interact with and inhibit PAP, which implies that snRNP-bound U1A would not be inhibitory.

However, previous work on the 3′ end processing of bovine and human papillomavirus (BPV and HPV) transcripts had shown that U1 snRNP itself was capable of suppressing 3′ end processing by binding to a 5′ ss and inhibiting polyadenylation [40]. In this case, though direct PAP inhibition was mediated by a separate U1 snRNP component, U1-70 K (Figure 1(c)), through a domain containing related basic-residue motifs, suggesting a similar mechanism is involved [41]. Unlike U1A, U1-70 K contains multiple basic-residue motifs and thus is self-sufficient in repressing PAP. Like in the case of free U1A, U1 snRNP can also in some contexts (e.g., in HIV RNA regulation) inhibit cleavage from a downstream position [42, 43]. Overall, these key observations implied that U1snRNP itself might play a more general inhibitory role in polyadenylation, *via* U1-70 K. Indeed, natural or mutated U1 snRNP targeted at the 3′ UTR of reporter [44] or endogenous genes [45] show strong inhibitory effects on polyadenylation, leading to robust downregulation of target genes. Tethering the PAP-interacting domain of U1-70 K (Figure 1(d)) is sufficient to mediate inhibition [46], whereas nearby tethering of SR proteins (which would engage U1-70 K) interferes with the inhibition, supporting the proposed model. More recently, the powerful effect of tethering U1 snRNP to block polyadenylation was also demonstrated in *trans *(Figure 1(e)), by the use of adaptor bifunctional modified oligonucleotides [47]. Importantly, this strategy introduced the concept that this mechanism can be harnessed as an antisense tool for gene-silencing, with relevant therapeutic perspectives, which will be discussed below.

## 5. U1 snRNP-Dependent IPA Suppression: A Safeguard of Transcript Integrity and an NMD Companion

Overall, the studies illustrated above highlighted a clear splicing-independent role for U1 snRNP in suppression of 3′ end processing in the context of PAS contained within a canonical 3′ UTR.

However, PAS contain limited amount of information, as well as sequences that could potentially work as PASs, but are never or seldom used, are very abundant in pre-mRNAs, in particular within introns [15, 17]. Usage of these putative PAS and would lead to significantly shortened mRNAs either unable to express functional proteins or expressing truncated variants, with potentially deleterious effects. It stands to reason that a suppressive mechanism must have evolved to minimize aberrant widespread IPA.

**Figure 2:**
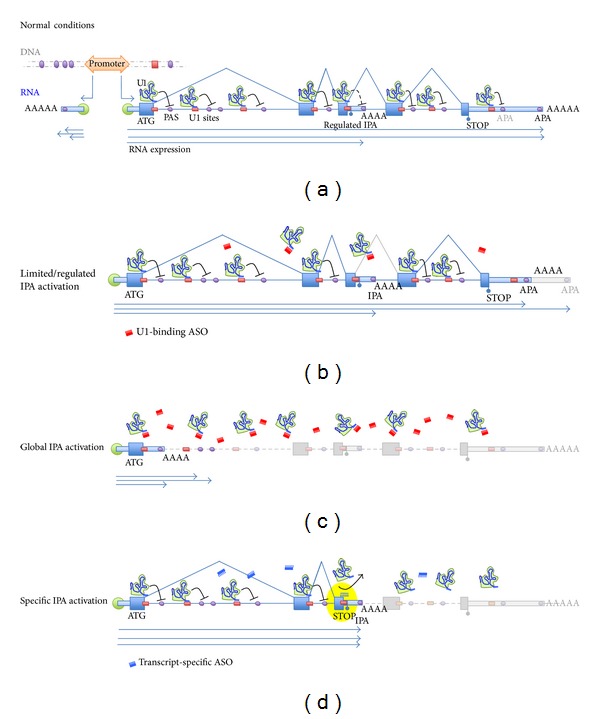
U1 snRNP suppression of cleavage and polyadenylation safeguards transcriptome integrity. (a) In normal conditions, transcription starts bidirectionally from a RNAPII promoter. Relative enrichment of PAS and depletion of U1 sites leads to short nonproductive mRNAs on the antisense strand, thus enforcing promoter directionality (described in Almada et al. 2013 [49]). In the sense strand, the presence of U1-binding motifs at splice sites and along introns recruits U1 snRNP and suppresses IPA, allowing full-length expression. Regulated IPA sites can still be expressed depending on cellular context. (b) Reduction of U1 snRNP activity to levels still compatible with efficient splicing by partial functional knockdown using U1-binding ASOs (described in Vorlová et al. [19]) or following changes in endogenous levels [50], leads to selective APA. PASs in stronger context (or suppressed by weaker U1 sites) are activated first. These likely include regulated IPA sites and tandem APA sites, a situation that reflects what may occur in proliferative/cancer cells, with the appearance of shorter average mRNAs in part due to relative shortage of available U1 snRNP. (c) Complete disruption of U1 activity by sequestering ASOs leads to loss of splicing and release of global IPA activation. Because of transcriptional directionality, earlier IPA sites get used first, resulting in massive shortening of mRNAs (described in Kaida et al. [48]). (d) When ASOs targeted to a specific 5′ ss are used, U1 binding is disrupted in that particular location but still functions normally elsewhere. The result is the selective activation of the targeted IPA site (highlighted), with expression of a truncated variant (described in Vorlová et al. [19]). Expression of other genes is not disrupted. U1 snRNP is indicated, as well as U1-targeting ASOs (red) and transcript-specific ASOs, (blue). Large blue and light blue boxes indicate exons and UTRs, respectively, while lines depict introns. PAS are depicted as purple ovals, U1 sites as red bars. Blue arrows indicate mRNA species generated in a specific context.

In its canonical splicing function, U1 snRNP is located at the 5′ ss of each intron, from where it could also inhibit downstream activation of IPA sites, much in the way by which tethered U1 snRNP blocks polyadenylation within a proper 3′ UTR (Figure 2(a)). Two recent studies directly tested this hypothesis and proposed an expanded role for U1 snRNP in 3′ end processing inhibition, which would put U1 at the center of a novel RNA surveillance mechanism that safeguards the integrity of pre-mRNAs from improper usage of premature PAS and that modulates the usage of legitimate IPA sites [19, 48].

Functional knockdown of U1 activity can be achieved with decoy RNA oligonucleotides directed against the 5′ end of the U1 snRNA, which in high concentration lead to the block of the splicing reaction [51]. When the effects of functional U1 depletion using high concentration of morpholino antisense compounds (ASOs) were assayed on a genomic tiling array [48], the generation of shorter stable pre-mRNA transcripts was observed in addition to splicing inhibition (Figure 2(c)). In these transcripts, cleavage and polyadenylation had occurred prematurely, typically within the first intron. Importantly, they were not activated when splicing was pharmacologically inhibited, or when U2 snRNP activity was abrogated [48]. In a parallel study [19], similar decoy RNAs were used to impair U1 snRNP functionality in conditions where splicing was preserved (Figure 2(b)). Under these conditions, a general activation of natural alternative IPA sites occurred and was recapitulated by siRNA knockdown of U1-70 K [19]. When ASOs targeted at specific 5′ ss upstream of the natural alternative IPA were used to prevent U1 snRNP binding there (rather than targeting U1 snRNA and preventing U1 from binding anywhere), only that specific IPA site was activated [19] (Figure 2(d)). Furthermore, inhibition of the same splicing event by blocking the downstream 3′ ss with a separate ASO does not activate IPA, while blocking the PAS directly with another ASO impedes activation of the truncated transcript [19], demonstrating that both the 5′ ss and the intronic PAS are essential. Together, these two studies show that U1 snRNP ability to inhibit 3′-end processing is not limited to the 3′ UTR but it extends to the entire pre-mRNA transcript, indicating a broad surveillance role for U1.

The range of U1's inhibitory effect appears to be limited to ~1 Kb [50], suggesting that the positioning of U1 at its natural 5′ position would not be sufficient to silence IPA events along large introns. Rather, its binding to the abundant pseudo 5′ ss that “litter” introns [52] might be functionally important to extend the protection from improper 3′ end processing to more distal areas (Figure 2(a)). Indeed, evidence of such binding of U1 to pseudosites was recently provided, as functional down-titration of cellular U1 snRNP levels with ASOs resulted in the *directional*  3′ → 5′ release of suppression of APA, whereas the opposite was observed following U1 snRNA overexpression [50].

The role of U1 snRNP as a safeguard of transcript integrity is also reflected in its recently described essential role in ensuring promoter directionality [49]. In fact, while RNAPII transcription is inherently bidirectional, transcripts in the antisense direction are shortly terminated by the presence of immediate PAS. On the contrary, the asymmetric enrichment of U1 snRNP-binding sites in the sense strand ensures that early PAS are suppressed and transcription is allowed to proceed productively.

In summary, U1 snRNP, in addition to its role in AS regulation, appears to play a nonsplicing key role in ensuring proper gene expression, by acting as an essential safeguard of the integrity of all transcripts. Moreover, it also complements the mRNA surveillance functions carried out by the conserved nonsense-mediated decay (NMD) machinery, which monitors and tags for degradation mRNAs harboring premature termination codons (PTC) within their ORF and that potentially encode for deleterious truncated proteins (reviewed in [53]). Typically, PTCs are generated by direct nonsense mutations or by frameshifts due to insertions, deletions, and aberrant splicing. In mammalian cells, NMD recognizes PTCs by their position upstream of the last exon-exon junction during translation and targets them for degradation. However, this leaves a significant gap in the mRNA surveillance process, as equally deleterious mRNA products could be generated from activation of cryptic intronic PAS. Such aberrant mRNAs would be effectively immune to NMD and evade degradation, since an activated intronic PAS would generate a novel “terminal exon”, with likely a new STOP codon located beyond the “new last” exon-exon boundary (if no in-frame new termination codons were present, the mRNA would be degraded by the nonstop mRNA decay pathway, reviewed in [54]). The U1 snRNP-mediated RNA surveillance function described above, would therefore, also protect from potential damage derived from mutations/lesions that introduce *de novo* PAS in introns.

Finally, these observations have additional far-reaching implications for the interpretation of disease-associated mutations. For example, mutations within 5′ ss, typically predicted to affect splicing and/or induce exon skipping, could in fact activate downstream intronic poly (A) sites, with the consequent generation of stable and potentially pathogenic truncated variants. Importantly, such events would likely be underestimated, as they would not be predicted based on DNA sequencing data, nor would they be detected by routine PCR-based analysis.

## 6. Modulation of U1 snRNP Functions as a Potential Cancer Therapy

The role of U1 snRNP in ensuring the integrity of pre-mRNA through suppression of polyadenylation can also be harnessed in novel antisense-based strategies to control gene expression. The use of next-generation antisense oligonucleotides is emerging as effective approaches to treat many conditions, including genetic diseases and cancer (reviewed in [55]) and can be similarly used to modulate U1 snRNP function to develop therapeutic approaches to target such diseases.

## 7. U1 Adaptors to Knockdown Tumorigenic Pre-mRNA Transcripts

The unique features of U1 snRNP in suppression of 3′ end processing described above constitute the foundation of a novel powerful gene-silencing technology, U1 small nuclear interference (U1i). This technology uses bifunctional oligonucleotide “U1 adaptors” to recruit U1 to the UTR of endogenous pre-mRNAs (Figure 1(e)) to suppress their polyadenylation and target them for degradation by the 3′-5′ exosome [47].

2-O-Methyl (2′OMe) or locked nucleic acids (LNA) oligonucleotides are designed to contain a 5′ targeting moiety, specific to the 3′ UTR of the target transcript, coupled to a 3′ U1-binding moiety, which is similar to a 5′ ss and base-pairs to the U1 snRNA [47]. Hybridization of the U1 adaptor oligonucleotide to the target transcript results in recruitment of U1 snRNP, which in turn inhibits nuclear poly(A) polymerase activity through U1-70K, leading to RNA degradation.

Recently, in an important proof-of-concept study, the U1 adaptor approach was shown to be highly effective in suppression of melanoma growth in xenografts models, by targeting metabotropic glutamate receptor 1 (GRM1) and B-cell lymphoma 2 (BCL2) transcripts [56]. Aberrant expression of GRM1, a transmembrane domain G protein-coupled receptor that mediates glutamate signaling, plays a crucial role in the onset of melanoma in mouse models [57], and dysregulated glutamatergic signaling leads to transformation and tumorigenesis in multiple cancer types [57, 58]. The antiapoptotic gene BCL2 is a frequent player in a variety of cancers, providing an escape mechanism to avoid the effects of apoptosis-inducing chemotherapeutic compounds [59]. U1 adaptors were delivered to tumors by a dendrimer RGD delivery system which binds with high affinity to an integrin variant overexpressed on the surface of many solid tumors [60]. Systemic delivery of the U1 adaptors targeting BCL2 and GRM1 suppresses tumor growth in melanoma xenografts by up to 60–70% [56].

In a separate study, U1 adaptors were targeted to PIM1 kinase (proviral integration site for Moloney murine leukemia virus 1), a constitutively active serine/threonine kinase that regulates a diverse array of cellular responses, including apoptosis and cell signal transduction [61]. PIM1 overexpression in multiple tumor types is linked to poor prognosis. *In vitro* inhibition of PIM-1 by siRNAs was previously shown to mediate antitumorigenic effects in colorectal and prostate carcinoma cells [62]. U1i adaptors targeted to PIM1 effectively and specifically silence PIM1 in GBM cell lines, with antiproliferative and proapoptotic effects [61]. Similarly, PIM1-U1i adaptors, encapsulated within nanoparticles and injected intratumorally into glioblastoma xenografts, reduced tumor growth by 40–50%, compared to controls.

Off-target and nonspecific effects, observed in other antisense approaches such as RNAi, could also represent an issue with U1i technology. In particular, a possible pitfall of U1i is its potential to nonspecifically sequester endogenous U1 snRNP, an occurrence that can in fact induce global changes in splicing and processing of nontargeted transcripts [63]. However, this should become evident only at very high concentration of U1i adaptors, whereas the high-potency compounds developed by Goraczniak and colleagues are used at concentrations that should not significantly affect global U1 snRNP functions.

Collectively, these studies demonstrate that U1i technology, by exploiting the ability of U1 snRNP to inhibit 3′ polyadenylation and hence targeting a transcript for degradation by the 3′–5′ exosome, provides a viable approach for effective knockdown of tumorigenic transcripts *in vivo*.

## 8. Activation of Antitumorigenic Isoforms by Release of U1 snRNP-Mediated Suppression of Intronic PAS

As described above, treatment of cells with decoy ASOs mimicking 5′ ss inhibits U1 snRNP functions globally and nonspecifically, and leads to global activation of normally suppressed PAS [19, 48]. However, ASOs that instead compete with endogenous U1 snRNP for specific 5′ ss within target transcripts can be used to induce the activation of specific natural IPA events [19]. Often, the truncated proteins generated by the activation of the APA event lack important functional C-terminal regions and possess dominant-negative properties.

In absence of an actionable downstream IPA site, splicing redirection ASOs would interfere with the splicing reaction, typically leading to exon skipping [55]. Antisense modulation of splicing events with this class of compounds is emerging as a viable therapeutic approach to induce more desirable splicing variants in genetic diseases such as Duchenne muscular dystrophy or spinal muscular atrophy and has reached the clinical trial stage (reviewed in [64]). In cancer, a leading approach has been to induce antagonist/dominant negative variants of oncogenic proteins (reviewed in [55]), for example, in the case of signal transducer and activator of transcription 3 (STAT3), where redirection of its splicing to the antitumorigenic Stat3 beta variant leads to full tumor regression in breast cancer mouse models [65]. A similar approach was adapted to activate antitumorigenic IPA variants in receptor tyrosine kinase genes, to induce the expression of secreted decoy RTK (sdRTK) isoforms that antagonize RTK signaling [19].

Deregulation and constitutive activation of RTK signaling represents a key aspect of tumorigenesis in a broad range of human cancers [66, 67]. Targeting these oncogenic pathways has provided the basis for targeted therapies, with the development of effective tyrosine kinase inhibitors (TKI) or antibodies directed at the extracellular domains (ECD) [68]. An alternative approach to inhibit oncogenic RTK signaling has been the delivery of recombinant sdRTKs variants, composed of the ligand-binding ECD [69]. In this context, signaling is blocked *via* ligand sequestration and/or the engagement of endogenous full-length RTKs in nonproductive dimers. This is the basis for how Aflibercept, a VEGF trap, functions to effectively suppress neovascularization and tumor growth [70].

**Figure 3:**
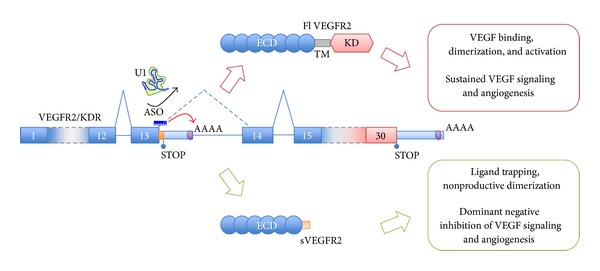
Therapeutic potential of IPA activation: induction of secreted decoy VEGFR2. An IPA site in intron 13 of VEGFR2 can be specifically and effectively activated using ASOs targeted to the 5′ ss immediately upstream, preventing U1 from binding and thus releasing suppression [19]. This leads to the expression of a variant secreted decoy VEGFR2 encoding the sole ECD. This variant can still bind VEGF or other ligands and can still dimerize with VEGF receptors, but it cannot signal. On the contrary, it leads to a blockade of VEGF signaling in targeted and surrounding cells, with dominant negative properties.

Usage of IPA sites upstream of the exons encoding for the transmembrane domain of RTKs, to generate sdRTK, was recently shown to commonly occur in most RTK mRNAs [19]. Treatment with ASOs coupled to a dendrimer delivery moiety, to target the 5′ ss upstream of the IPA site, resulted in its activation and expression of specific endogenous sdRTKs isoforms for multiple RTKs, including *EGFR*, *MET*, *HER2*, *VEGFR1*, and *VEGFR2* [19]. *VEGFR2* is the major mediator of VEGF signaling and a central player in tumor vascularization [71]. As such, anti-VEGF treatment is a cornerstone of cancer therapy, with drugs targeting both the VEGF ligand (bevacizumab and aflibercept) as well as its receptor (sunitinib and axitinib). As mentioned, soluble VEGFR2 is a powerful natural inhibitor of angiogenesis [26] and is underrepresented in tumors [27]. Its induction by activation of a PAS in intron 13 of the VEGFR2/KDR pre-mRNA (Figure 3) resulted in the generation of a soluble protein isoform that potently inhibited angiogenesis in a paracrine and autocrine fashion [19] and also showed activity *in vivo* [72].

Given the central role of aberrant RTK signaling in cancer and the existence of sdRTK variants for most RTKs, their induction by the specific, U1 snRNP-competing, ASO-mediated activation of IPA has a tremendous potential as a broad therapeutic approach in cancer therapy.

## 9. Conclusion and Future Directions

Our understanding of the role of U1 snRNP in pre-mRNA processing has gradually expanded from its initial splicing functions in splice-site selection and exon definition to its moonlighter act in the suppression of polyadenylation in a very limited, gene-specific manner to the currently proposed role as a key player in RNA surveillance and global safeguard of mRNA integrity (and possibly long noncoding RNAs) against spurious cleavage and polyadenylation, with a job description that has come to include also the control of promoter directionality in transcription.

Much still remains to be uncovered about the specific mechanisms underlying how U1 snRNP manages to effectively wear its many hats. However, the compendium of evidence outlined above points to a basic model where U1 snRNP associates with the CTD of RNAPII and is then recruited cotranscriptionally to the nascent transcript at multiple sites throughout the length of the pre-mRNA. These correspond to either genuine or pseudo 5′ ss and collectively result in the suppression of cleavage and polyadenylation along the pre-mRNA, enforcing its full-length expression (Figure 2(a)).

Genuine PAS utilization is achieved by the evolutionary depletion of U1-binding sites in the UTR and/or by the presence of dominant cis-acting regulatory elements that potentiate PAS or inhibit U1 activity through specific factors. For example, any factor that promotes recruitment of U1 snRNP to a splice site would strengthen IPA inhibition and *vice versa*. Therefore, in certain contexts, like in the case of VEGFR2 and other RTKs, the system has evolved past the default suppressive status, in order to allow specific APA events to occur.

In general, proliferative/cancer cells demonstrate a global shortening of 3′ UTRs, typically resulting in enhanced gene expression. U1 snRNP might be indirectly contributing to this phenomenon if the increase in transcription—and the related increase in associated machinery, including cleavage and polyadenylation factors—is not paralleled by an equivalent increase in U1 snRNP levels. The relative depletion of U1 snRNP would release PAS and could in part explain the observed directional effect, as shown in activated neurons [50]. This selective switch from distal to proximal PAS can be countered *in vitro* via overexpression of U1 snRNA [50], and it is, thus, possible to envisage an approach to promote 3′ UTR re-lengthening by ectopic expression of U1 snRNA by gene therapy technologies. Alternatively, since 3′ end processing can be specifically blocked by ASO directed at the PAS [19], transcript-specific ASO could be employed to selectively block the usage of proximal PAS while reactivating distal ones.

Overall, the possibility to harness U1 snRNP-mediated suppression of polyadenylation has created an attractive and still mostly unexplored opportunity to reshape the transcriptome for therapeutic purposes, in cancer and other diseases. U1i technology provides an effective alternative approach to siRNA *in vivo*, and at the same time the activation of sdRTKs by IPA derepression could serve as a blueprint for the induction of therapeutically relevant, endogenous, potent dominant-negative IPA variants of oncogenes. A better molecular understanding of the role of U1 snRNP in APA is an essential step in the design of rationally targeted antisense strategies as effective therapies.
